# Express delivery logistics with high-speed railway: a perspective of payment scheme and forecast information sharing

**DOI:** 10.1007/s40747-021-00304-1

**Published:** 2021-03-18

**Authors:** Huawei Duan, Yusen Ye, Zheng Lei, Mengting Wang

**Affiliations:** 1 School of Management, Xihua University, Chengdu, 610031 Sichuan China; 2 Institute for Disaster Management and Reconstruction, Sichuan University, Chengdu, 610017 Sichuan China

**Keywords:** Express delivery logistics, Advance payment, Forecast information sharing

## Abstract

This paper investigates the payment scheme and forecast information sharing issues in the express delivery logistics with the high-speed railway. The HSR carriers need to coordinate the transportation capacity between passenger and freight. It is widely recognized that the advance payment scheme (APS) using as deposit is a beneficial way for the HSR carriers to make decisions on the transportation capacity preserved for express delivery. However, the express service providers, who possess private forecast information of express delivery demand, may share inaccurate information with the HSR carriers to acquire sufficient preserved transportation capacity. This paper discusses what payment scheme is preferred by the HSR carrier, the express service provider through discussing the deposit decisions with or without forecast information sharing. We show that sharing demand forecast information can reduce the prereserved capacity and increase the profits of the HSR carrier. With the delayed payment scheme (DPS), the express service provider has no motivation to share the information; while with the APS, the HSR carrier can reasonably choose the deposit to encourage the express service provider to share the demand information. Our analysis also shows that the HSR carrier’s profits with the APS is restricted by the investment returns and the express service provider’s information sharing decisions. We also analyze the value range of the deposit, which is a proportion of the overall payment, that allows both the HSR carrier and the express service provider to prefer the APS, as well as to encourage the express service provider to share the demand information.

## Introduction

Over the past decade, the quantity of express delivery parcels in China has climbed up from 1.9 billion pieces to 64 billion pieces. With the rapid development of e-commerce and online grocery, the market demand of express delivery continues surging. Even during the massive quarantines in case of the outbreak of COVID-19 pandemic, the express delivery business has surprisingly risen by 11.5% over the same period of the last year. In contrast, the shortage of express transportation capacity is always the bottleneck in response to the cost and time efficiency required by express industry [1]. The average intercity delivery time of a parcel excesses 3 days. Even for one of the busiest lines of express business, from Beijing to Shanghai, it generally needs more than 24 h to ensure the delivery of a parcel. To improve the shipping performance between some main cities, one of the leading express service providers in China, the SF express, has cooperated with the China Railway Express that provides speedy express delivery service within 10 h by utilizing the surplus capacity of Fuxing bullet passenger trains running along the Beijing–Shanghai high-speed railway. As the network scale of high-speed railways expands across the nation, the high-speed rail express delivery (HSReD) becomes preferred by the express service providers who accommodate time-sensitive delivery services. Shipping with mid-long distances (i.e., 1,000–2,400 km), HSReD has superior time efficiency and punctuality rate than air transportation owing to quicker cargo security check and minor impacts of bad weather and traffic control. The HSReD capacity of SF express was soon fully loaded after the delivery service was launched with 100% punctuality rate. Meanwhile, HSReD can offer considerable shipping capacity. During the “double 11” shopping festival last year, the overall transportation volume of HSReD reached 2,650 tons, which effectively mitigated the overloaded stress of express service providers. Compared to shipping by air or by road, HSReD is also competitive in terms of lower transportation cost, less economic investments, smaller environmental impacts and lower energy consumption [2]. For instance, the mainline transportation cost in China of HSReD is 25% less than air express delivery, while the energy consumption per cargo turnover and the carbon emission per shipping unit by high-speed rail are respective 1/7 and 1/13 of those by road. As the advantages of HSReD has been increasingly recognized by practitioners, express delivery services have grown up as the core business of high-speed rail (HSR) carriers. The 2019 annual revenue of HSReD has increased 15%, ranking top of the business of the China Railway Express.

To take full advantage of the transportation capacity, the HSR carriers need to coordinate the space between passenger and freight. There are four mainstream carrying modes adopted for HSReD, i.e., using the dynamic inspecting train, sharing the spare space of passenger carriages, transforming passenger carriages into freight carriages and operating specialized high-speed freight trains. All these carrying modes require HSR carriers to evaluate the transportation capacity prereserved for express delivery services. In the face of uncertain express market demand and potential competition with other transportation modes (i.e., air or road transportation), the HSR carriers prefer to contract with the express service providers before the selling season to pre-determine the transportation volume. In practice, the Chinese HSR carriers, such as the China Railway Express, ask the express service providers to pay a deposit, which is a proportion of the overall freightage, for the transportation capacity preservation. By pulling the demand risk with express service providers, it is a beneficial way for the HSR carriers to better organize the shipping operations for express freight in coordination with passenger carrying services. However, while the HSR carriers expect to increase the deposit to shift the demand risk, a high deposit may force the express service providers to quit the cooperation and use other transportation modes for express delivery. How much should the express service providers pay as a deposit for transportation capacity preservation? How does the deposit decision relate to the preserved transportation capacity decision? We are interested in these questions.

On the other hand, information structure plays a vital role in the trade-off problems. When the express service providers are equipped with private information about the demand, forecast information sharing will help the HSR carriers make more accurate decisions on the preservation of transportation capacity for express freight. However, the express service providers, who share the demand risk with the HSR carriers, are possibly reluctant to share the demand information in contracting, depending on the deposit they paid. If the overall freightage is paid afterwards, it is difficult to guarantee that the express service providers would share real-demand information [3]. What decision should the HSR carriers make to encourage the express service providers to share real-demand information? What payment scheme is beneficial for the HSR carrier, the express service provider? These important decision-making issues, to the best of our knowledge, have very few discussions in the existing literature.

In this paper, we will investigate what payment scheme is preferred by the HSR carrier, the express service provider through discussing the deposit decisions with or without forecast information sharing. Our model is based on the classic newsvendor model and we assume that the HSR carrier and the express service provider play a Stackelberg game. By giving the thresholds of the deposit, we first analyze the impact of advance payment scheme (APS) on the preservation decision of transportation capacity and discuss the conditions that encourage the express service providers to voluntarily accept the APS. Then, we analyze the value of information sharing to HSR carriers, express service providers with APS and show the conditions under which the express service providers would voluntarily share the demand forecast information.

This paper contributes to the state of the art research in three ways. First, as the HSReD has become competitive in the express delivery market, the revenue management of express delivery logistics from the perspective of payment scheme has not been emphasized by the literature. Second, the APS is considered as a beneficial way for the HSR carriers to better organize the shipping operations for express freight in coordination with passenger carrying services. However, a high deposit may force the express service provider to turn to air transportation carriers. We determine the optimal deposit decisions that encourage the express service provider to voluntarily accept the APS. Third, we investigate the role of demand forecast information sharing in the decision-making of the transportation capacity preservation and the deposit, which is a critical issue but has not yet been well explored in the relevant literature.

The rest of the paper is organized as follows. We review the relevant literature in the next section. We introduce the model framework and build the basic model with delayed payment scheme (DPS) in the third section and discuss the equilibrium results with APS in the fourth section. Based on our equilibrium results with two payment schemes, we discuss the respective conditions for the HSR carrier and the express service providers to adopt APS, and the express service providers to share the demand information. The sixth section is the numerical study and in the last section, we summarize our conclusions and provide additional managerial insights for additional discussions.

## Literature review

The present study is correlated to the previous work on revenue management in HSReD logistics. At present, some scholars have begun to carry out theoretical research on China’s HSReD logistics: Liu et al. [4] put forward the market positioning and product series of HSReD, in combination with the development status of China’s HRS carrier. Based on Stackelberg game theory, Duan et al. [5] and Lyu et al. [6] construct the pricing game model of HSReD supply chain under the stochastic market demand. Bi et al. [2] analyze the adaptability of HSReD to the HSR network according to the capacity utilization ratios of various HSR lines. These above studies mainly focused on the design and pricing of HSReD products, and the adaptability of HSReD. As far as it is known, few studies have investigated capacity reservation decision, in relation to the HSReD supply chain. However, capacity reservation and allocation has been widely considered in related industries [7, 8], such as the air-cargo industry [9]. In the case that the market demand is determined by the efforts of freight forwarders, and the efforts of freight forwarders cannot be verified, Gupta [10] designed two kinds of capacity contracts. Hellermann et al. [11] designed the option contract in the case of freight forwarder overbooking. The optimal booking strategy was drawn, and the impact of overbooking on airline profits was analyzed. Tao [12] designed an option contract with reservation cost and execution cost, in the case of competition among multiple freight forwarders, and analyzed the impact of competition among freight forwarders on contract design. All these above-mentioned literatures investigated the design of transportation volume allocation contract under the condition of symmetric information, adopting the DPS. However, we study on the capacity reservation when HSR carriers adopt the APS under the condition of asymmetry information.

Inventory management with the Advance Payment Scheme is also a popular research topic in recent years. Taleizadeh [13] investigated EOQ models allowing multiple prepayments scheme instead of a single prepayment option. Then, proposed an advance payment related inventory model for an evaporating item. Zia and Taleizadeh [14] extended the previous models by taking the concept of both advance payments and delayed payment. Lashgari et al. [15] proposed an inventory model using financial considerations under two levels credit policy. Taleizadeh [16] established an advance payment related lot-sizing model along with planned backordering. In a recent study, Khan et al. [17] studied a two-warehouse inventory model under multiple advance payments with partial backordering. Chang et al. [18] investigated optimal pricing and lot-sizing decisions for perishable products when the supplier demands that the manufacturer use a combination of advance, cash, and credit payments for the total purchase cost. Currently, Taleizadeh et al. [19] proposed an EOQ model with mixed sales when the payment scheme is mixed with multiple advance payment and partial credit payment. However, all of these papers studied the inventory model with advance payments under the condition of symmetric information.

Research on supply chain information sharing has been very rich. Previous studies have concentrated on the incentive to share information and the value of information. The bulk of the extant literature on this topic has investigated how information sharing affects pricing decisions within a supply chain [20–27]. Another stream of research on this topic has investigated inventory-related issues in a supply chain where asymmetric demand information exists [28–32]. Recently, scholars have explored the incentive of information sharing in some special supply chain and analyzed the impact of the information sharing. Mittendorf et al. [33] find that it benefits not only the retailer but also the manufacturer and consumers when the retailer chooses to share its information in a supply chain which the manufacturer undertakes services to increase product demand. Zhang et al. [34] find that the retailer prefers to share the information with the manufacturer on the condition of highly cost efficiency for the manufacturer’s after-sale service, when the manufacturer undertakes the after-sale service. Liu et al. [35] show that information sharing cooperation is more likely to occur when the supplier is more economical in terms of freshness-keeping investment, or when the e-tailer is more efficient in terms of service investment in a fresh produce supply chain. Lai [36] find that the forecast sharing from the port in a maritime supply chain can not only improve the profits for both parties, but also facilitate the sustainability investment, even when the carrier is risk averse. Nazifa et al. [37, 38] and Ongus et al. [39] explore the role of information sharing and using information technology from empirical study. However, none of those literature have concentrated on how information sharing affects the capacity reservation decision with APS.

The present study is more closely related to the literature focus on the capacity reservation decision in a supply chain where asymmetric demand information exists. Özalp Özer [3] has concentrated on the supplier’s capacity decision in the supply chain that supplier is responsible for acquiring the necessary capacity before receiving an order from the manufacturer who possesses private forecast information for her end product. They develop two contracts to enable credible forecast information sharing, and address how different contracts affect the supplier’s capacity decision. Mishra [40] examine the incentives and the value of demand forecast sharing in different production strategies such as Make-to-Order and Make-to-Stock. They analyze the production quantity decision in make-to-stock scenario that manufacturer sets the production quantity before the demand is realized. They show that if the savings from inventory holding and shortage costs because of information sharing are sufficiently high, then a side payment contract that induces Pareto-optimal and information sharing is feasible in the make-to-stock scenario. Especially, Amaruchkul [41] focus on the capacity contract between a carrier and a forwarder in air-cargo supply chain when certain parameters such as the forwarder’s demand, operating cost to the carrier, margin, and reservation profit are forwarder’s private information. They propose contracts in which the forwarder pays a lump sum in exchange for a guaranteed capacity allotment and receives a refund for each unit of unused capacity according to a pre-announced refund rate. Above literatures show that the manufacturer who possesses private forecast information does not have the incentive to share information although forecast information share benefits the supplier. Therefore, they all have concentrated on developing contracts to enable credible forecast information sharing.

All these above-mentioned literatures realized information sharing through the relevant design of supply chain contracts. The present focus the optimal deposit decision, which makes express service providers voluntarily share the real-demand forecast information. If the feasibility can be demonstrated in theory, this would provide an economical and easy way for both sides to realize information sharing.

## Model framework

Considering a HSReD supply chain that comprises of a HSR carrier selling transport service at a agreed freight rate *w* to an express service provider, who sells the express product to consumers at the fixed market price *p*. HSR carrier is responsible for acquiring the necessary capacity before receiving an order from the express service provider who possesses private forecast information for end express product. HSR carrier reserves high-speed rail transport capacity *Q* with the unit capacity reservation cost *C*_Q_, and provides high-speed rail express service with unit volume service cost *c*.

It was assumed that $p > w > c + c_{Q}$. Otherwise, the high-speed rail express will not make profit. In addition, when the market demand is less than the reserve capacity, the unused reserve capacity does not generate inventory cost, and the value of the remaining reserve capacity may not be temporarily considered. When the market demand is greater than the reserve capacity, it is assumed that the penalty cost of the unsatisfying market demand is 0.

The sequence of events is as follows: (1) when the two sides establish a cooperative relationship, HSR carrier determines the agreed freight rate *w* and the proportion of the overall freightage paid in advance (1-*t*), express service provider forecasts market demand and determines whether to share information with HSR carrier; (2) in the HSReD planning period, HSR carrier determines the reserve capacity *Q*, express service provider need to pay part of the freight in advance according to (1-*t*); (3) in the sales period of HSReD, the market demand *D* is clear, express service provider orders the actual needed high-speed rail express service according to the market demand *D*, HSR carrier provides service which is not greater than the reserve capacity *Q*. After the completion of the service, express service provider needs to pay the remaining freight.

### Demand function

The demand function was set as $D = \mu + \varepsilon$, where: $\mu$ refers to the market size; $\varepsilon$ represents the randomness of the market demand, which is a random variable with normal distribution, with a mean value of 0 and a variance of $\sigma^{2}$. Assuming that the express service provider can obtain a private demand forecast information $\gamma$, $\gamma = \varepsilon + \xi$, where: $\xi$ captures the noise in the forecast error, which is a random variable of normal distribution, with a mean value of 0 and a variance of $\sigma_{0}^{2}$. According to an existing literature [36, 40], it was concluded that $E[\varepsilon \left| \gamma \right.] = \frac{{\sigma^{2} \gamma }}{{\sigma^{2} + \sigma_{0}^{2} }}$, $\sigma_{\gamma }^{2} {\text{ = Var}}[\varepsilon \left| \gamma \right.] = \frac{{\sigma^{2} \sigma_{0}^{2} }}{{\sigma^{2} + \sigma_{0}^{2} }}$.

### Basic model (DPS)

To compare this with the APS, the present study initially constructs the basic game model and analyze the equilibrium with DPS. DPS refers to the payment of the express service provider for the freight, according to the actual transportation volume after the express transportation task was completed by HSR carrier. In fact, DPS is a special form of the APS. That is, when the deposit is 0, it is easier to implement, since there is no need to consider the setting of the deposit. Hence, this has been widely used in aviation and maritime transportation.

With DPS (*D*), express service provider often only pays the cost of the actual transportation volume after the completion of the express service. The loss caused by part of the reserve capacity, which is greater than the actual market demand, is borne by HSR carrier.

With DPS (*D*), the function of expected profit of the HSR carrier (*R*) is

1 $$ E[\pi_{D}^{R} ] = (w - c)E{\text{min}}(Q_{D} ,D) - c_{Q} Q_{D} . $$

In this function, $(w - c)E{\text{min}}(Q_{C} ,D)$ represents the income from the service actually provided by HSR carrier, and $c_{Q} Q_{C}$ represents the cost of reserve capacity.

The function of expected profit of express service provider (E) is

2 $$ E[\pi_{D}^{E} ] = (p - w)E\min (Q_{D} ,D). $$

Based on the above profit function, it is easy to get Lemma 1.

#### Lemma 1

With DPS (*D*), the optimal reserve capacity of HSR carrier and the expected profit of both parties are as follows:

(i) Under the condition that express service provider does not share information (DN), the optimal reserve capacity of HSR carrier is

3 $$ Q_{{{\text{DN}}}}^{*}  = \mu { + }\sigma \Phi^{ - 1} \left( {\frac{{w - c - c_{Q} }}{w - c}} \right), $$

the profits of HSR carrier and express service provider are, respectively,

4 $$ \pi_{{{\text{DN}}}}^{R*} = (w - c - c_{Q} )\mu - \sigma (w - c)L(\sigma k), $$

5 $$ \pi_{{{\text{DN}}}}^{E*}  = (p - w)\mu + \sigma \frac{{(p - w)c_{Q} k}}{w - c} - \sigma_{\gamma } (p - w)L(\sigma k). $$

(ii) Under the condition that express service provider shares information (DY), the optimal reserve capacity of HSR carrier is

6 $$ Q_{\rm DY}^{*}  = \mu { + }\sigma_{\gamma } \Phi^{ - 1} \left( {\frac{{w - c - c_{Q} }}{w - c}} \right), $$

the profits of HSR carrier and express service provider are, respectively,

7 $$ \pi_{\rm DY}^{R*} = (w - c - c_{Q} )\mu - \sigma_{\gamma } (w - c)L(\sigma_{\gamma } k), $$

8 $$ \pi_{{{\text{DY}}}}^{E*} = (p - w)\mu + \sigma_{\gamma } \frac{{(p - w)c_{Q} k}}{w - c} - \sigma_{\gamma } (p - w)L(\sigma_{\gamma } k), $$

in which $k = \Phi^{ - 1} \left( {\frac{{w - c - c_{Q} }}{w - c}} \right)$, $L(x) = \int_{ - \infty }^{x} {(x - z)} d\Phi (z)$, and $\Phi$ is the probability distribution function of the standard normal distribution.

## Analysis of the APS (A)

With APS, HSR carrier needs to set a reasonable deposit, and determine the reserve capacity, to maximize its own profits, on the premise of ensuring that the express service provider participates in the cooperation. It is noteworthy that the HSR carrier uses the deposit in the investment, and obtains the corresponding investment returns. In the case of HSR carrier collects deposit in advance, and uses these for investment, the expected profit function of HSR carrier (R) is

9 $$ E[\pi_{A}^{R} ] = (tw - c)E\min (Q_{A} ,D) + I(1 - t)wQ_{A} - c_{Q} Q_{A} . $$

Hence, $({1} - t)wQ_{A}$ is the deposit collected in advance in the capacity reservation stage, and $twE\min (Q_{A} ,D)$ is the freightage collected after the completion of the service. In particular, with APS, HSR carrier will use the deposit for the investment. Assuming that the rate of return on investment is *i,* and for convenience, set *I* = 1 + *i*, then $I(1 - t)wQ_{A}$ would be total value of the prepaid expenses including the investment income.

The expected profit function of express service provider (E) is

10 $$ E[\pi_{A}^{E} ] = (p - tw)E\min (Q_{A} ,D) - (1 - t)wQ_{A} . $$

Hence, with APS, the express service provider needs to pay the deposit $({1} - t)wQ_{A}$ in the stage of capacity reservation.

Based on the above-mentioned profit function, the game equilibrium analysis was carried out for the HSReD supply chain with APS under the conditions of sharing information and not sharing information.

### No information sharing (N)

Since the express service provider does not share information, HSR carrier can only determine the reserve capacity $Q_{{{\text{AN}}}}$ based on the information on $\varepsilon$ shared by both parties. By substituting the demand function *D* into the profit function of HSR carrier, the following can be obtained:

11 $$ E[\pi_{{{\text{AN}}}}^{R} ] = (tw - c)[\int_{ - \infty }^{{Q_{\rm AN} - \mu }} {(\mu + \varepsilon )f(\varepsilon )} {\text{d}}\varepsilon + \int_{{Q_{\rm AN} - \mu }}^{ + \infty } {Q_{{{\text{AN}}}} f(\varepsilon ){\text{d}}\varepsilon } ] + I(1 - t)wQ_{{{\text{AN}}}} - c_{Q} Q_{{{\text{AN}}}} . $$

By solving the above formula, we can get Lemma 2.

#### Lemma 2

When express service provider does not share demand forecast information (*N*), the optimal reserve capacity is as follows:

12 $$ Q_{{{\text{AN}}}}^{*}  = \mu { + }\sigma \Phi^{ - 1} \left( {\frac{{I(t)w - c - c_{Q} }}{tw - c}} \right). $$

The profits of HSR carrier and express service provider is as follows:

13 $$ \pi_{\rm AN}^{R*} = (I(t)w - c - c_{Q} )\mu - \sigma (tw - c)L(\sigma k(t)), $$

14 $$ \pi_{\rm AN}^{E*}  = (p - w)\mu + \sigma \left[ {p - w - \frac{{(p - tw)(I(t)w - c - c_{Q} )}}{tw - c}} \right]k(t) - \sigma_{\gamma } (p - tw)L(\sigma k(t)), $$

where $I(t) = I(1 - t) + t$, $k(t) = \Phi^{ - 1} \left( {\frac{{I(t)w - c - c_{Q} }}{tw - c}} \right)$, $L(x) = \int_{ - \infty }^{x} {(x - z)} d\Phi (z)$, and $\Phi$ is a probability distribution function of the standard normal distribution.

In Lemma 2, $Q_{\rm AN}^{*}$ is divided into two parts. One part is the reserve capacity $\mu$, according to the determined market demand, while the other part is the reserve capacity $\sigma \Phi^{ - 1} \left( {\frac{{I(t)w - c - c_{Q} }}{tw - c}} \right)$ to cope with the randomness of the market demand. The smaller the prepayment proportion 1 − *t* is, the smaller the reserve capacity becomes, which is in line with the actual situation of high-speed rail express operation.

The profit of HSR carrier $\pi_{\rm AN}^{R*}$ is also divided into two parts. That is, $(I(t)w - c - c_{Q} )\mu$ represents the profit obtained by meeting the determined market demand, and $\sigma (tw - c)L(\sigma k(t))$ represents that the profit loss of HSR carrier, when the market demand is less than the reserve capacity.

The profit of the express service provider $\pi_{AN}^{E*}$ can be divided into three parts. The first part $(p - w)\mu$ represents the profit obtained in meeting the determined market demand; The second part $\sigma \left[ {p - w - \frac{{(p - tw)(I(t)w - c - c_{Q} )}}{tw - c}} \right]k(t)$ represents the influence of the reserve capacity for HSR carrier on the profits of the express service provider. The reserve capacity of HSR carrier is positively correlated with the profits of the express service provider, when $t[Ip + (1 - t)(I - 1)w - c - c_{Q} ] > Ip - c - \frac{{pc_{Q} }}{w}$ is satisfied. Otherwise, this is negatively correlated with the profits of the express service provider. The third part $(p - tw)\sigma_{\gamma } L(\sigma k(t))$ represents to the profit loss of express service provider when the market demand is less than the reserve capacity.

### Information sharing (*Y*)

When the express service provider shares its private demand forecast information $\gamma$, HSR carrier determines the reserve capacity according to the information on $\varepsilon$ that is shared by both parties, and the private prediction information $\gamma$ of the express service provider. In substituting the demand function *D* into the profit function of HSR carrier, the following can be obtained:

15 $$ \begin{aligned} E[\pi_{\rm AY}^{R} \left| \gamma \right.] & = (1 - t)wQ_{\rm AY} + (tw - c)E\min (Q_{\rm AY} ,D) - c_{Q} Q_{\rm AY} \\ & = (tw - c)E\left\{ {\left[ {\int_{ - \infty }^{{Q_{\rm AY} - \mu }} {(\mu + \varepsilon )f(\varepsilon )} d\varepsilon + Q_{\rm AY} \int_{{Q_{\rm AY} - \mu }}^{ + \infty } {f(\varepsilon )d\varepsilon } } \right]\left| \gamma \right.} \right\} + (w - tw - c_{Q} )Q_{\rm AY} . \\ \end{aligned} $$

By solving the above formula, Lemma 3 can be got.

#### Lemma 3

When express service provider shares information (Y), the optimal reserve capacity is as follows:

16 $$ Q_{\rm AY}^{*}  = \mu { + }\sigma_{\gamma } \Phi^{ - 1} \left( {\frac{{I(t)w - c - c_{Q} }}{tw - c}} \right), $$

the profits of HSR carrier and express service provider are as follows:

17 $$ \pi_{\rm AY}^{R*} = (I(t)w - c - c_{Q} )\mu - \sigma_{\gamma } (tw - c)L(\sigma_{\gamma } k(t)), $$

18 $$ \pi_{\rm AY}^{E*} = (p - w)\mu + \sigma_{\gamma } \left[ {p - w - \frac{{(p - tw)(I(t)w - c - c_{Q} )}}{tw - c}} \right]k(t) - \sigma_{\gamma } (p - tw)L(\sigma_{\gamma } k(t)), $$

where $I(t) = I(1 - t) + t$, $k(t) = \Phi^{ - 1} \left( {\frac{{I(t)w - c - c_{Q} }}{tw - c}} \right)$, $L(x) = \int_{ - \infty }^{x} {(x - z)} d\Phi (z)$, and $\Phi$ is the probability distribution function of the standard normal distribution.

### Analysis on value range of $t$

According to Lemmas 2 and 3, the feasible scope of *t* that indicates the proportion of the overall freightage paid after the service is completed is preliminarily analyzed. According to the value range of the probability distribution function in formula (13), the following can be obtained: $tw - c > 0$. Hence, $t > 1 - \frac{{c_{Q} }}{Iw}$. In addition, *t* is the proportion and the following can be obtained: $0 \le t \le 1$. In conclusion, $1 - \frac{{c_{Q} }}{Iw} \le t \le 1$.

Furthermore, (1-*t*) indicates the proportion of the overall freightage paid in advance, and it should not exceed $\frac{{c_{Q} }}{(1 + i)w}$. That is, the max value of (1-*t*) is determined by the capacity reservation cost $c_{Q}$, the agreed freight rate $w$, and the rate of return on investment *i*. When $t = 1$ ($1 - t{ = 0}$), HSR carrier will not charge any deposit when determining the reserve capacity, the APS is equivalent to the DPS in essence.

## Comparison and analyses

### Comparison of two payment scheme

By comparing the relationship between the optimal reserve capacity and the profits of both parties with DPS and APS, the influence of payment scheme on the reserve capacity and profits of both parties can be obtained, and the value range of *t* that makes both parties accept the APS can be further determined. Refer to Result 1 and 2 for the details.

**Result 1.** In the case of information sharing and no information sharing of express service provider, the relationship between the optimal reserve capacity with DPS and APS is as follows: $Q_{\rm AY}^{*} > Q_{\rm DY}^{*}$_,_ $Q_{\rm AN}^{*} > Q_{DN}^{*}$.

It can be observed that regardless of whether express service provider shares demand forecast information, the reserve capacity with APS will be greater than that with DPS. With APS, the deposit made by express service provider can offset the cost for the reserve capacity, reducing the risk of profit loss of HSR carrier. Therefore, with APS, HSR carrier is willing to reserve more capacity for the express service provider, which can reduce the loss caused by the shortage of reservation.

According to Lemmas 1, 2 and 3, the conditions for availability of $\pi_{\rm AY}^{R*} \ge \pi_{\rm DY}^{R*}$, $\pi_{\rm AY}^{E*} \ge \pi_{\rm DY}^{E*}$ can be concluded through the detailed analysis, and the value range of *t* that makes HSR carrier and express service provider both accept the APS could be worked out. Refer to Result 2 for the details.

**Result 2.** When express service provider shares information, if *t* meets the following conditions, this can make HSR carrier and express service provider obtain more profits with APS:

(i) When *t* meets the following condition:

19 $$ (I(t) - 1)wu - \sigma_{\gamma } w[tM{ + }(t - 1)L(\sigma_{\gamma } k))] - \sigma_{\gamma } cM \ge 0, $$

the profit obtained by HSR carrier with APS is greater than that with DPS, that is, HSR carrier adopts APS.

(ii) When *t* meets the following condition:

20 $$ p(K - Q - M) - w[K - t(Q + M) + (1 - t)(k\Phi (k){ + }L(\sigma_{\gamma } k))] \ge 0, $$

the profit obtained by express service provider with APS is greater than that with DPS, that is, express service provider accepts APS.

(iii) When *t* meets the following condition:

21 $$ p(K - Q - M){ + }w\left\{ (I(t) - 1)u - K - t[Q + (1 - \sigma_{\gamma
} )M] + (1 - t)[k\Phi (k){ + }(1 - \sigma_{\gamma }
)L(\sigma_{\gamma } k)]\right\} - \sigma_{\gamma } cM \ge 0, $$

the profits of whole supply chain with APS are both greater than those with DPS.

(iv) When the conditions in (i) and (ii) are met at the same time, the profits of HSR carrier and express service provider with APS are both greater than those with DPS, that is, APS is superior to DPS, where $M = L(\sigma_{\gamma } k(t)) - L(\sigma_{\gamma } k)$, $Q = k(t)\Phi (k(t)) - k\Phi (k)$,$K = k(t) - k$.

**Result 3.** When express service provider does not share information, if *t* meets the following conditions, this can make HSR carrier and express service provider obtain more profits with APS:

(i) When *t* meets the following condition:

22 $$ (I(t) - 1)wu - \sigma w[tN{ + }(t - 1)L(\sigma k))] - \sigma cN \ge 0, $$

the profit obtained by HSR carrier with APS is greater than that with DPS, that is, HSR carrier adopts APS.

(ii) When *t* meets the following condition:

23 $$ p(\sigma K - \sigma Q - \sigma_{\gamma } N) - w[\sigma K - t(\sigma Q + \sigma_{\gamma } N) + (1 - t)(\sigma k\Phi (k){ + }\sigma_{\gamma } L(\sigma k))] \ge 0, $$

the profit obtained by express service provider with APS is greater than that with DPS, that is, express service provider accepts APS.

(iii) When *t* meets the following condition:

24 $$ p(\sigma K - \sigma Q - \sigma_{\gamma } N){ + }w\{ (I(t) - 1)u - \sigma K + t[\sigma Q + (\sigma_{\gamma } - \sigma )N] - (1 - t)[\sigma k\Phi (k){ + }(\sigma_{\gamma } - \sigma )L(\sigma k)]\} - \sigma cN \ge 0, $$

the profits of whole supply chain with APS are both greater than those with DPS.

(iv) When the conditions in (i) and (ii) are met at the same time, the profits of HSR carrier and express service provider with APS are both greater than those with DPS, that is, APS is superior to DPS.

Where $$N = L(\sigma k(t)) - L(\sigma k)$$, $$Q = k(t)\Phi (k(t)) - k\Phi (k)$$,$$K = k(t) - k$$.

### Comparison of no information sharing and information sharing

Initially, the present study analyzed the incentive of information sharing of express service provider with DPS. According to Lemma 1, it is easy to obtain Result 4.

**Result 4.** With DPS, in the cases of information sharing (*Y*) and no information sharing (N), the relationship between the reserve capacity and the profits of both parties is as follows: $Q_{\rm DY}^{*} < Q_{DN}^{*}$, $\pi_{\rm DY}^{R*} > \pi_{DN}^{R*}$, $\pi_{\rm DY}^{E*} < \pi_{DN}^{E*}$.

It can be observed that when express service provider shares information, this can improve the accuracy of the demand forecast of HSR carrier, reduce capacity reservation, and increase the profits of HSR carrier. However, HSR carrier reduce the reserved capacity, the risk that reserved capacity cannot meet the market demand increase, thereby reducing the profits of express service provider. Therefore, with DPS, express service provider will not voluntarily share the information. In existing research, information sharing was based on contract implementation.

Next, this analyzed the incentive of information sharing of express service provider with APS. According to Lemmas 2 and 3, Result 5 can be obtained.

**Result 5.** With APS, and in the case of information sharing (Y) and no information sharing (N), the relationship between the reserve capacity and profits of both parties is as follows: $Q_{\rm AY}^{*} < Q_{\rm AN}^{*}$, $\pi_{\rm AY}^{R*} > \pi_{\rm AN}^{R*}$.

It can be observed that with APS, sharing information can reduce the reserve capacity of HSR carrier, and increase its profits. However, the profits of the express service provider under information sharing and non-sharing, which are $\pi_{\rm AY}^{R*}$ and $\pi_{\rm AN}^{R*}$, were correlated to *t.* Therefore, with APS, HSR carrier can realize information sharing by adjusting the proportion of advance payment. The following presents the specific analysis of the value range of *t*, which can encourage the express service provider to voluntarily share information.

According to Lemmas 1 and 2, the value of information sharing to HSR carrier $v^{R}$, the value to the express service provider $v^{E}$, and the value to the supply chain $v$ can be defined as follows:

25 $$ v^{R} = \pi_{\rm AY}^{R*} - \pi_{\rm AN}^{R*}  =  = (tw - c)[\sigma L(\sigma k(t)) - \sigma_{\gamma } L(\sigma_{\gamma } k(t))], $$

26 $$ v^{E} = \pi_{\rm AY}^{E*} - \pi_{\rm AN}^{E*} { = (}\sigma_{\gamma } - \sigma )[p - w - (p - tw)\Phi (k(t))]k(t) - \sigma_{\gamma } (p - tw)G, $$

27 $$ v = v^{E} + v^{R} { = (}\sigma_{\gamma } - \sigma {)[}p - w - (p - tw)\Phi (k(t)]k(t) - \sigma_{\gamma } (p - c)G - (\sigma_{\gamma } - \sigma )(tw - c)]L(\sigma k(t)), $$

where $$G = L(\sigma_{\gamma } k(t)) - L(\sigma k(t))$$.

For further analysis of conditions that make $v^{R} > 0$,$v^{E} \ge 0$, Lemma 4 can be concluded.

**Lemma 4**. (i) When *t* meets the following conditions, $v^{R} > 0$:

28 $$ 1 - \frac{{c_{Q} }}{Iw} \le t \le 1, $$

(ii) When *t* meets the following condition, $v^{E} \ge 0$:

29 $$ [p - w - (p - tw)\Phi (k(t))]k(t) \le \frac{{\sigma_{\gamma } }}{{\sigma_{\gamma } - \sigma }}(p - tw)G, $$

(iii) When *t* meets the following condition, $v \ge 0$:

30 $$ [p - w - (p - tw)\Phi (k(t))]k(t) \le \frac{{\sigma_{\gamma } }}{{\sigma_{\gamma } - \sigma }}(p - c)G{ + }(tw - c)L(\sigma k(t)). $$

According to Lemma 4, the relationship between the value range of *t* and the information sharing decision of express service provider is detailed in Result 6.

**Result 6.** When *t* meets the following conditions, demand forecast information sharing can increase the profits of HSR carrier, the express service provider, and the supply chain.

(i) When *t* meets the basic conditions for the optimal reserve capacity, the value of information sharing to HSR carrier $v^{R}$ will be greater than 0. That is, when express service provider shares its private demand information, this can increase the profits of HSR carrier.

(ii)When $[p - w - (p - tw)\Phi (k(t))]k(t) \le \frac{{\sigma_{\gamma } }}{{\sigma_{\gamma } - \sigma }}(p - tw)G$, $v^{E}$ will not be less than 0. That is, when the express service provider shares its private demand information, this could increase (at least not reduce) its own profits. At this time, express service provider will have incentive to share information.

(iii) When $\frac{{\sigma_{\gamma } }}{{\sigma_{\gamma } - \sigma }}(p - tw)G < [p - w - (p - tw)\Phi (k(t))]k(t) \le \frac{{\sigma_{\gamma } }}{{\sigma_{\gamma } - \sigma }}(p - c)G{ + }(tw - c)L(\sigma k(t))$, $v^{E}$ will be less than 0, but the value to the supply chain $v$ will be greater than 0. That is, when the express service provider shares its private demand information, this would reduce its own profits, but in turn, increases (at least not reduce) the overall profit of the supply chain. At this time, express service provider has no incentive to share information, but HSR carrier can promote express service provider to share information through Side Pay.

(iv) When $[p - w - (p - tw)\Phi (k(t))]k(t) > \frac{{\sigma_{\gamma } }}{{\sigma_{\gamma } - \sigma }}(p - c)G{ + }(tw - c)L(\sigma k(t))$,$v$ will be less than 0. That is, when express service provider shares its private demand information, this would reduce the overall profit of the supply chain. At this time, HSR carrier can make the express service provider share information only through the implementation of the supply chain contract.

## Impact of payment scheme and information sharing

The reserve capacity and expected profits of both parties with different payment schemes and information sharing strategies can be calculated, the influence of payment scheme and information sharing can be analyzed, and the range of *t* that can make HSR carrier and express service provider accept APS and encourage the express service provider to share information can be further determined. According to the actual operation data of the high-speed rail express [5, 6, 27, 42], the relevant parameters were set as follows: $\mu { = 100}$, $\sigma { = 2}$, $\sigma_{{0}} { = 1}$, $p = 20$, $w = 12$, $c = 6$, $c_{Q} = 2$, and $I = {1}{\text{.1}}$, according to the feasible scope of *t* concluded in the present study, set $t \in [0.85,{1}]$.

### Impact of payment scheme

#### Impact of payment scheme on HSR carrier

**Fig. 1:**
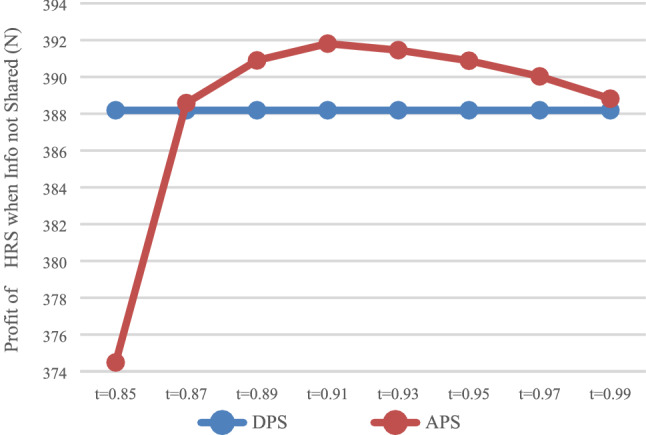
Impact of payment scheme on HSR (N)

**Fig. 2:**
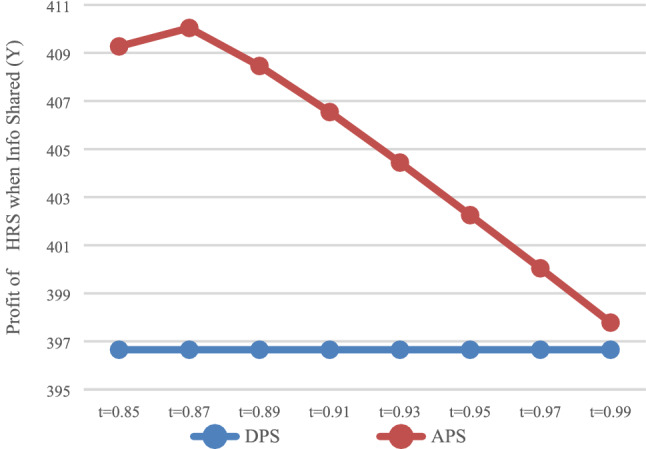
Impact of payment scheme on HSR (Y)

The expected profit of HSR carrier was calculated with DPS (D) and the APS (A), respectively, and the influence of the payment scheme on the profit of HSR carrier was analyzed. In the case of information that was not shared (N), the values of $\pi_{DN}^{R*}$ and $\pi_{\rm AN}^{R*}$ is shown in Fig. 1, while in the case of information that was shared (Y), the values of $\pi_{\rm DY}^{R*}$ and $\pi_{\rm AY}^{R*}$ are shown in Fig. 2.

It can be observed that when express service provider does not share information (Fig. 1), HSR carrier may not necessarily obtain more profits with APS, but would instead damage its own profits when it charges a higher proportion of prepayment fees. When express service provider shares information (Fig. 2), HSR carrier can obtain more profits with APS.

#### Impact of payment scheme on express service provider

**Fig. 3:**
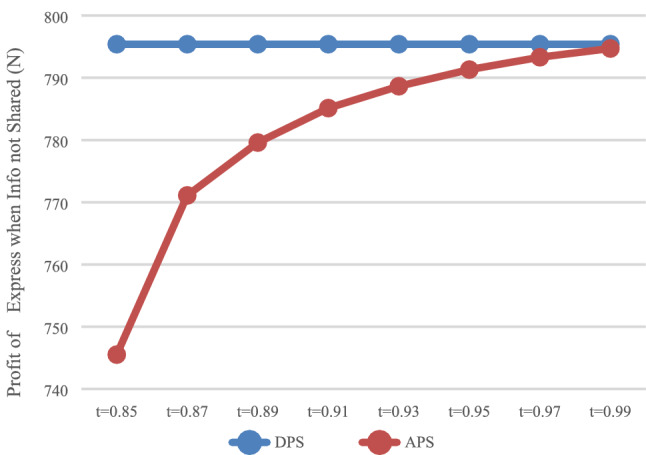
Impact of payment scheme on express (*N*)

**Fig. 4:**
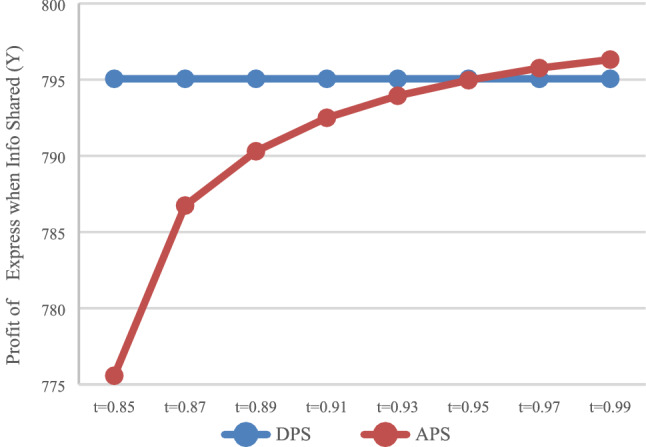
Impact of payment scheme on express (*Y*)

The expected profit of the express service provider with DPS (D) and APS (A) can be calculated. In the case of no information sharing (N), the values of $\pi_{DN}^{E*}$ and $\pi_{\rm AN}^{E*}$ are presented in Fig. 3. In the case of information sharing (Y), the values of $\pi_{\rm DY}^{E*}$ and $\pi_{\rm AY}^{E*}$ are presented in Fig. 4.

It can be observed that when information is not shared (Fig. 3), the express service provider makes smaller profits with APS. That is, when express service provider does not share information, it will not voluntarily accept advance payments. In the case of information sharing (Fig. 4), that is, when $t \in [0.97,0.99]$, express service provider makes greater profits with APS, and this would allow them to voluntarily accept the advance payment.

#### Impact of payment scheme on reserve capacity

**Fig. 5:**
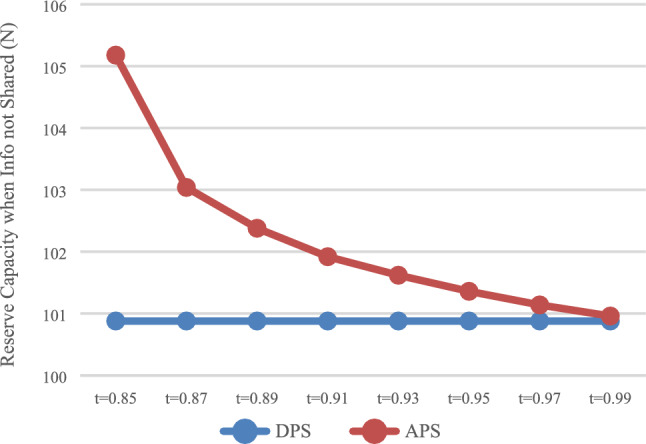
Impact of payment scheme on capacity (N)

**Fig. 6:**
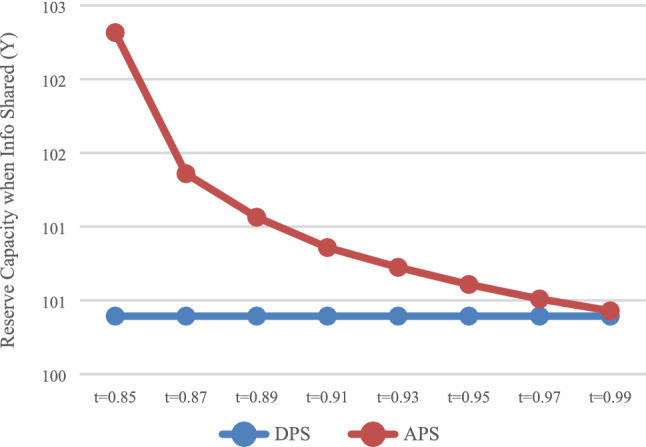
Impact of payment scheme on capacity (Y)

The reserve capacity with DPS (D) and APS (A) was calculated, respectively, and the influence of the payment scheme on reserve capacity was analyzed. In the case of no information sharing (N), the values of $Q_{DN}^{*}$ and $Q_{\rm AN}^{*}$ are presented in Fig. 5. In the case of information sharing (Y), the values of $Q_{\rm DY}^{*}$ and $Q_{\rm AY}^{*}$ are presented in Fig. 6.

As shown in Figs. 5 and , that is, regardless of whether the express service provider shares information, HSR carrier will reserve more capacity with APS, which is consistent with the conclusion of Result 1.

### Impact of information sharing

#### Impact of information sharing on profits of both enterprise

**Fig. 7:**
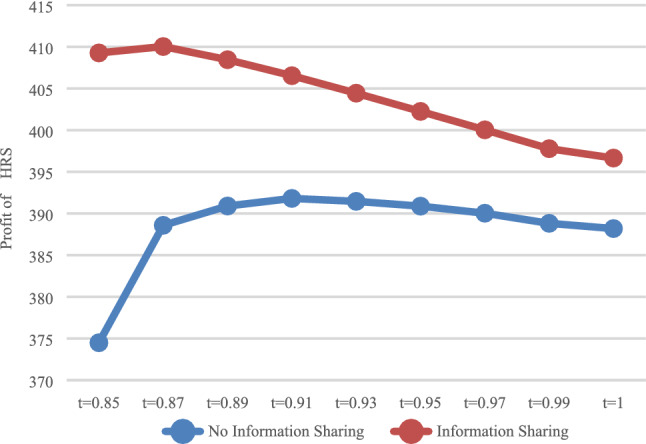
Impact of information sharing on profits of HSR

**Fig. 8:**
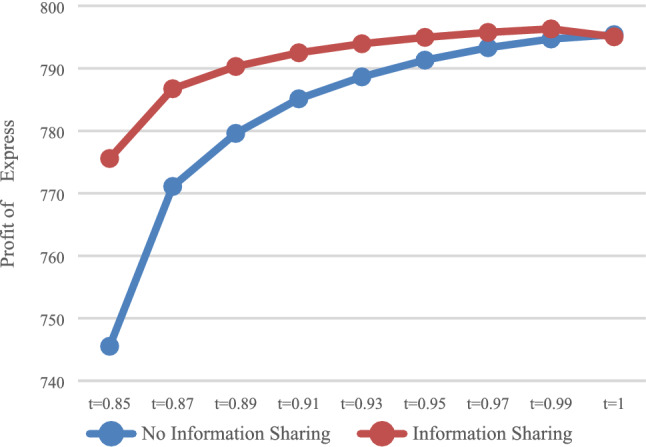
Impact of information sharing on profit of express

The expected profit of HSR carrier and express service provider were calculated in the case of no sharing information (*N*) and sharing information (*Y*), respectively. The expected profit of HSR carrier $\pi_{\rm AN}^{R*}$ and $\pi_{\rm AY}^{R*}$ are presented in Fig. 7, while that of express service provider $\pi_{\rm AN}^{E*}$ and $\pi_{\rm AY}^{E*}$, these are presented in Fig. 8.

As shown in Fig. 7, as long as the express service provider shares information, regardless of whether it is with DPS (*t* = 1) or The APS (*t* < 1), HSR carrier can obtain more profits, which is consistent with the conclusions of Results 4 and 5. For express service provider (Fig. 8), with DPS (*t* = 1), the profit when sharing information is smaller. That is, sharing information would lead to profit loss, while with The APS (*t* < 1), by setting the deposit within the appropriate scope, express service provider can obtain more profits when sharing information. That is, express service provider can be encouraged to voluntarily share information by reasonably setting the proportion of advance payment.

#### Impact of information sharing on reserve capacity

**Fig. 9:**
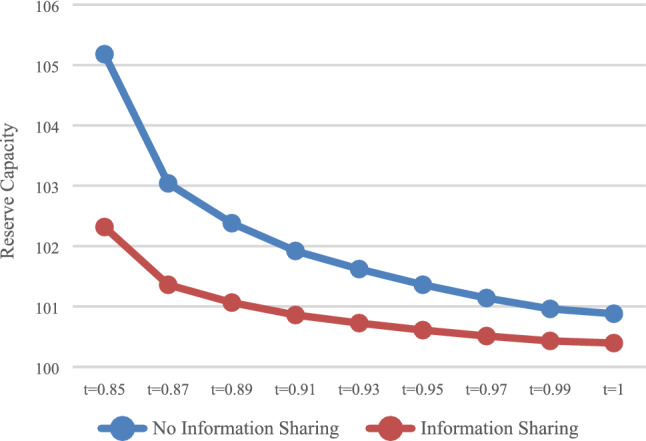
Impact of information sharing on reserve capacity

The optimal reserve capacity $Q_{\rm AN}^{*}$ and $Q_{\rm AY}^{*}$ were calculated under the condition of no information sharing (N) and information sharing (Y), respectively, as shown in Fig. 9.

Regardless of whether it was with DPS or APS, as shown in Fig. 9, when express service provider shares information, reserve capacity would be smaller, which is consistent with Result 4 and 5.

### The impact of rate of return on investment

**Fig. 10:**
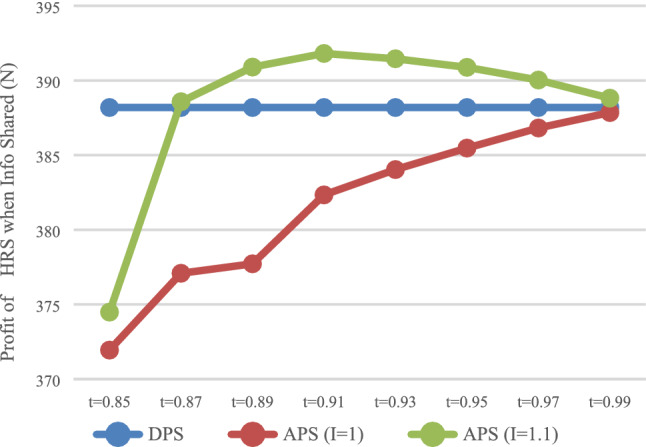
Impact of investment on HRS (N)

**Fig. 11:**
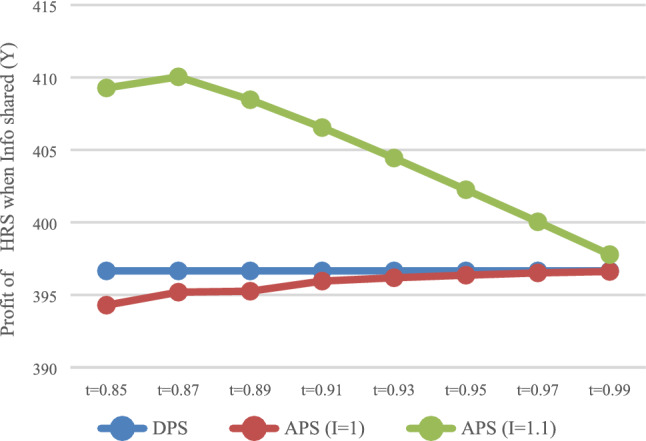
Impact of investment on HRS (Y)

If HSR carrier does not invest after collecting the fees in advance, the rate of return on investment is 0, that is, $I = {1}$. Under the condition of no information sharing (*N*), the profit of HSR carrier with DPS, the profit of HSR carrier with DPS when HSR carrier does not invest (*I* = 1) and the return on investment is 0.1 (*I* = 1.1), are shown in Fig. 10. The profits of HSR carrier in above three situation under the condition of information sharing (Y) are shown in Fig. 11.

As shown in Fig. 10, when the rate of return on investment is 0, the profit of HSR carrier with The APS is smaller than that with DPS. That is to say, if HSR carrier does not use the advance payment to invest, DPS would be more favorable for it. It can be observed that the key advantage of the APS is that HSR carrier can place the prepayment into the investment.

### Discussion on the value range of *t*

As shown in Figs. 1 and 2, when $t \in [0.87,0.99]$, HSR carrier would adapt APS when express service provider shares or does not share information. As shown in Fig. 3 and 4, when $t \in [0.{9}7,0.99]$, express service provider would accept APS under the condition of sharing information. It can be concluded that the value range of *t* for both parties to accept APS is $t \in [0.{9}7,0.99]$.

As shown in Fig. 8, when $t \in [0.8{5},0.99]$, the profits of express service provider when sharing information are greater than those when it does not share information. It can be concluded that the value range of *t*, which can encourage express service provider to share information, is $t \in [0.85,0.99]$.

In summary, the value range of *t* for express service provider to voluntarily share information, and for both parties accept APS is $t \in [0.{9}7,0.99]$.

## Conclusions

In the present study, a game model of the high-speed rail express supply chain was constructed with DPS and APS, when express service provider shares and does not share demand forecasting information. Then, the influence of the payment scheme and information sharing strategy on the reserve capacity and profits of both sides were analyzed by comparing the game equilibrium results. The following conclusions were drawn:

1. In terms of information sharing, with both payment schemes, the sharing of demand forecast information by express service provider can reduce the reserve capacity, and increase its profits of HSR carrier. For express service provider, there is no motivation to share information with DPS, but while with APS, a reasonable prepayment proportion can be set according to Results 6, in order to make it voluntarily share information.
2. In terms of the payment scheme, HSR carrier reserves more capacity with APS. However, whether these two parties accept APS depends on the setting of the deposit. It is noteworthy that HSR carrier may not be able to obtain more profits with APS, especially when express service provider does not share information and charge a higher proportion of prepayment. This is because HSR carrier will reserve more capacity with APS, and the decision-making error of reserve capacity caused by the inaccurate market forecast information may be greater, and the profit loss may also be greater. Therefore, when HSR carrier adopts APS, it should pay more attention to encourage information sharing from express service provider.
3. The key advantage of HSR carrier to adopt APS is that the deposit collected in advance can be used to investment. If HSR carrier does not invest, or the return on investment is too low, the APS may be unfavorable.

In summary, the present study shows that HSR carrier can reasonably set the deposit, allowing both sides to accept APS, and encouraging the express service provider to share information. The present also worked out the value range of the proportion of the overall freightage paid in advance under these above two circumstances, providing a theoretical basis for HSR carrier to promote the APS, and an economical and easy approach to realize the information sharing of both sides in express delivery logistics with the high-speed railway.

The present study investigates and analyzes the possibility of encouraging express service providers to share demand forecasting information by adjusting the deposit. However, express service providers also have private information, such as operating cost, margin, and reservation profit. Hence, information sharing decision-making and incentive problems can be further considered when express service providers have a variety of private information. In addition, the present study only considered the cooperation between one HSR carrier and one express service provider. Hence, the follow-up research should consider the information sharing and incentive problems under the situation of different types of express service providers participating in the cooperation.
